# A pleckstrin homology domain protein is involved in crystalloid formation in *Plasmodium* ookinetes and affects the maturation of infective sporozoites

**DOI:** 10.3389/fcimb.2026.1777159

**Published:** 2026-05-18

**Authors:** Mayumi Tachibana, Naoaki Shinzawa, Minami Baba, Motomi Torii, Richard Culleton, Tomoko Ishino

**Affiliations:** 1 Division of Parasitology, Proteo-Science Center, Ehime University, Ehime, Japan; 2 Department of Parasitology and Tropical Medicine, Institute of Science Tokyo, Tokyo, Japan

**Keywords:** crystalloid, ookinete, *Plasmodium*, pleckstrin homology (PH) domain, sporozoite invasion

## Abstract

The *Plasmodium* ookinete is a unique invasive stage which is formed following the fertilization of gametes in mosquito midguts after ingestion of infected mammalian blood. Unlike merozoites and sporozoites, ookinetes do not differentiate and proliferate inside cells; and rather penetrate the midgut epithelium and migrate through to the basal lamina, where they transform into oocysts. Sporozoites develop inside oocysts, and because sporozoites ultimately initiate malarial infections to humans, the prior ookinete stage is correspondingly a promising target to reduce malaria transmission. Ookinetes possess a distinctive crystalloid organelle which is named for its crystalline-like array of spherical particles. Recent research indicates that crystalloids influence the formation of oocysts and subsequent sporozoites, but the molecular mechanisms of the process are not understood. Here we focused on a novel crystalloid protein in *Plasmodium yoelii* that contains a pleckstrin homology (PH) domain. The protein is a paralog of the previously identified CryPH, and therefore we designated it PyCryPH2. Targeted disruption of the *pycryph2* gene resulted in an irregular microstructure of particles within the crystalloids. While *pycryph2*-deficient parasites developed into morphologically normal sporozoites, the parasites had a significantly reduced ability to invade mosquito salivary glands, as well as to infect mouse liver. These findings indicate that PyCryPH2 is involved in forming crystalloids with a well-organized internal architecture and contributes to the maturation of functional sporozoites either directly or through interaction with other crystalloid proteins.

## 1 Introduction

*Plasmodium* ookinetes form in the midgut of the *Anopheles* mosquito following the ingestion of parasite gametocytes during a blood meal, and pass through the midgut epithelium to differentiate into oocysts on the outer side of the midgut. The ookinete stage in the mosquito is a bottleneck in the malaria life cycle, and is therefore a major target for transmission-blocking strategies (Sinden, 2017). Within the mature ookinete to early oocyst, unique intracellular organelles called crystalloids are present, which are not observed in other stages. Electron microscopy has shown that the crystalloid forms as a cluster of characteristic spherical particles and is surrounded by hemozoin and vacuoles (Dessens et al, 2011). The LCCL lectin adhesive proteins (LAPs), which contain adhesive domains called LCCL (*Limulus* clotting factor C, Coch-5b2 and Lgl1), have been studied in detail as the major crystalloid-localized proteins (Claudianos et al, 2002; Raine et al, 2007; Carter et al., 2008; Ecker et al., 2008; Saeed et al., 2010, 2013; 2015, 2018). Reverse genetics studies in *Plasmodium berghei* have shown that deletion of LAP1 (PbSR) or LAP3 resulted in the absence of crystalloids (Carter et al., 2008; Saeed et al., 2015), and parasites deficient in any of the LAP family molecules were shown to have a significant inhibition of sporogony in oocysts (Claudianos et al., 2002; Raine et al., 2007; Ecker et al., 2008; Saeed et al., 2015). It has been reported that disruption of two enzymes localized to crystalloids, S-acyltransferase DHHC10 and NAD(P) transhydrogenase, also resulted in failure of both crystalloid and sporozoite formation (Santos et al., 2016; Saeed et al., 2020). These results suggest that proteins localized to crystalloids have an important influence on sporozoite formation (sporogony) in oocysts; however, the molecular mechanisms and specific roles remain poorly understood. Many of the proteins which have been implicated in crystalloid formation, such as the LAP and CPW-WPC protein families, are found across the apicomplexan phylum, and including *Cryptosporidium* and proto-apicomplexans such as *Chromera* and *Vitrella* (Rao et al., 2016; Tosini et al., 2004; Abrahamsen et al., 2004; Templeton and Pain, 2016). This suggests that any role suggested for the crystalloid must consider an ancient conserved function across alveolate protozoans.

In our investigation of ookinete-specific proteins in *Plasmodium yoelii*, we identified a novel molecule containing a pleckstrin homology (PH) domain that localizes to crystalloids and designated it as PyCryPH (PY17X_0705200) (Jenwithisuk et al., 2018). Subsequently, in *P. berghei* ookinetes, four PH domain-containing proteins, including an orthologue of PyCryPH, were reported to localize to the crystalloids (Tremp et al., 2020). The PH domain, a structural module of approximately 120 amino acids, is known to bind to specific phospholipids within the plasma membrane and is implicated in the intracellular signal transduction, membrane protein localization, and various physiological processes in cells (Lemmon, 2007; Powis et al., 2023). However, it has been reported that putative PH domains in apicomplexans share only weak homology with classic PH domains and possess a distinct amino acid signature, suggesting parasite-specific functions (Tremp et al., 2020). Nevertheless, the functions of PH-domain-containing molecules localized to crystalloids have not been analyzed.

In this study, we focused on PyCryPH2 (PY17X_0705100), previously designated as PyCryPH-p in Jenwithisuk et al, 2018, whose gene is adjacent to *pycryph* in the genome in a head-to-head orientation. PyCryPH2 possesses a PH domain and shares 20% identical amino acid residues with PyCryPH. By raising specific antibodies against PyCryPH2, we demonstrated that it also localizes to crystalloids in ookinetes. Its physiological functions during mosquito-stages were investigated by targeted gene disruption in *P. yoelii*. Our results revealed that PyCryPH2 is involved in the formation of characteristic spherical particle structures within the crystalloid; and its loss perturbs sporozoite maturation, resulting in a reduced ability to invade salivary glands and infection of mouse liver.

## 2 Materials and methods

### 2.1 Parasites and mosquitoes

To obtain blood stage parasites, cryopreserved *P. yoelii* 17XNL-infected blood was intraperitoneally injected into ICR female mice (Nippon CLEA, Tokyo, Japan) which were pretreated with 1.2 mg phenyl-hydrazine to induce reticulocyte production. Mice were kept at a room temperature of 24 °C under a 12 h light/12 h dark cycle. To enrich schizonts, infected mouse blood was collected by cardiac puncture and was layered onto 55% (v/v) Nycodenz (Sigma-Aldrich, St. Louis, MO, USA) in PBS solution and centrifuged at 450 × *g* for 20 min at room temperature. Parasites were collected on the interface and washed twice with ice-cold PBS containing protease inhibitors (PI, Roche Applied Science, Penzberg, Germany). For *in vitro* ookinete culture, infected mouse blood was collected by cardiac puncture. After passing through a CF11 (Whatman, Maidstone, UK) column to remove white blood cells, the infected erythrocytes were resuspended in ookinete culture medium (RPMI 1640 medium containing 20% heat inactivated fetal calf serum, 0.367 mM hypoxanthine, 25 mM HEPES, 24 mM NaHCO_3_ and 5 IU/ml heparin, pH8.4), and incubated at 24 °C for 16 h. The ookinetes were collected by density-gradient centrifugation as described above. For the production of mosquito stage parasites, *Anopheles stephensi* SDA500 mosquitoes were allowed to feed on anesthetized *P. yoelii* 17XNL*-*infected mice, and fully engorged female mosquitoes were selected and kept at 24 °C until dissection. At days 8, 12, 13, 14, and 16 post-feeding, midguts and salivary glands were collected by dissection to assay the numbers of oocysts and sporozoites, respectively. At day 13 post-feeding, hemolymph sporozoites were collected by infusion of 100 µl of RPMI 1640 medium (FUJIFILM Wako Pure Chemical, Osaka, Japan) through the mosquito thorax; to count the number of sporozoites and to examine their motility *in vitro*, as described in a following gliding motility section. Sporozoites were counted using mosquitoes obtained from cages with a mean oocyst intensity of 30 or more. For RNAseq analysis midgut sporozoites at day 13 were purified using a 17% (w/v) Accudenz solution (Accurate Chemical & Scientific Corporation, Carle Place, NY, USA) (Kennedy et al., 2012).

### 2.2 Antibody production

To obtain PyCryPH2 specific antibodies, a region with lower similarity compared to PyCryPH was selected to produce recombinant protein as antigen for immunization (Jenwithisuk et al., 2018). A fragment encoding PyCryPH2 (amino acid [aa] positions 289 to 386) was amplified by PCR from *P. yoelii* 17XNL genomic DNA, using PyCryPH2‐EcoRV‐F1 (5′‐tattttcagggcgatatcGCAAATTTTAAAATAAAAGGCAT‐3′) and PyCryPH2‐BamHI‐R1 (5′‐gcggtacccgggatccCTATAAATTATCATCATCATTATC‐3′). The amplified *pycryph2* DNA fragment was inserted between the EcoRV and BamHI sites of the plasmid vector, pEU-E01-GST-TEV-N1 (CellFree Sciences, Matsuyama, Japan). The recombinant PyCryPH2 was expressed fused with glutathione S-transferase (GST) tag at its N-terminus and was produced using the wheat germ cell-free protein synthesis system (CellFree Sciences). Recombinant protein was purified using a Glutathione Sepharose 4B column (GE Healthcare, Camarillo, CA, USA) and eluted with elution buffer (40 mM reduced glutathione, 50 mM Tris-HCl, 300 mM NaCl, 200 mM imidazole, 2% glycerol, pH 8.0).

To generate antibodies against PyCryPH2, a Japanese white rabbit was immunized subcutaneously with 250 μg of purified recombinant PyCryPH2 emulsified in Freund’s complete adjuvant, followed by two booster immunizations with 250 μg of purified recombinant PyCryPH2 with Freund’s incomplete adjuvant. Immunizations were performed at 3-week intervals and antisera was collected 14 days after the last immunization (Kitayama labes Co. Ltd., Ina, Japan).

### 2.3 Western blotting

Schizont, ookinete, and sporozoite proteins were extracted in reducing SDS-PAGE loading buffer and boiled at 97 °C for 5 min, followed by electrophoretic separation on a 12% polyacrylamide gel (ATTO, Tokyo, Japan). Proteins were transferred to a 0.2 μm polyvinylidene fluoride (PVDF) membrane (ATTO), which was incubated with Blocking One (Nacalai Tesque, Inc., Kyoto, Japan) followed by immunostaining with rabbit antiserum against PyCryPH2 (1:1,000 dilution) or PyCryPH (1:1,000 dilution, Jenwithisuk et al., 2018) as the primary antibody. The membranes were then probed with HRP-conjugated goat anti-rabbit IgG antibody (1:30,000 dilution, Promega, Madison, WI, USA) and visualized with Immobilon Western Chemiluminescent HRP Substrate (Merck Millipore, Billerica, MA, USA) on a LAS 4010 luminescent image analyzer or Amarsham ImageQuant800 (Cytiva, Malboorough, MA, USA). The relative molecular masses of the proteins were estimated with reference to Precision Plus Protein Standards (Bio-Rad, Hercules, CA, USA). Rabbit anti-PyCPWWPC1 antiserum (1:1,000 dilution) was used as a stage-specific loading control for ookinetes, and rabbit anti-PbRAMA antiserum (1:1,000 dilution) for schizonts and sporozoites (Kangwanrangsan et al., 2013; Ishino et al., 2019).

### 2.4 Indirect immunofluorescent analysis

Smears of *P. yoelii* 17XNL-infected blood containing schizonts and gametocytes were fixed on glass slides with 4% paraformaldehyde. Sporozoites from salivary glands were harvested at day 14 post-feeding on 8-well slides, and smears of cultured ookinetes were prepared as described above, and both were fixed with 4% paraformaldehyde. Slides were permeabilized with 0.1% Triton X-100 (Nacalai Tesque, Inc.), followed by blocking with PBS containing 5% fetal calf serum at 37 °C for 30 min. The slides were then incubated with rabbit anti-PyCryPH2 antiserum (1:100) or rabbit anti-PyCryPH antiserum (1:100) together with stage specific marker antibodies; specifically, mouse anti-Pys25 mAb (ookinete marker, 1:1,000), mouse anti-PyMSP1 antiserum (schizont marker, 1:10,000), mouse anti-PyG377 antiserum (female gametocyte marker, 1:100), or mouse anti-PyCSP antiserum (sporozoite marker, 1:100,000). Incubations were performed at 37 °C for 1 h, followed by incubation with Alexa Fluor 488-goat anti-rabbit IgG antibody and Alexa Fluor 546-goat anti-mouse IgG antibody (Invitrogen, Thermo Fisher Scientific, Carlsbad, CA, USA) as secondary antibodies (1:500 dilution) at 37 °C for 30 min, together with 1 μg/mL 4′,6-diamidino-2-phenylindole (DAPI) as a nuclear marker (Tsuboi et al., 1997; Otsuki et al., 2015; Ishino et al., 2020; Tachibana et al., 2024). After mounting in ProLong Gold antifade reagent (Invitrogen, Thermo Fisher Scientific), samples were observed using a fluorescence microscope (Axio Scope.A1, Carl Zeiss, Oberkochen, Germany).

### 2.5 Electron microscopy

For conventional transmission electron microscopy, *P. yoelii* 17XNL-infected erythrocytes containing gametocytes were fixed in 2% paraformaldehyde and 2% glutaraldehyde in HEPES-buffered saline (pH 7.05), washed in the same buffer, and post-fixed in 2% osmium tetroxide. The specimens were then dehydrated in a graded series of ethanol and embedded in Epon 812 resin (TAAB Laboratories Equipment Ltd, Aldermaston, UK). For immuno-transmission electron microscopy, *P. yoelii* 17XNL-infected erythrocytes containing gametocytes or transgenic parasite-infected erythrocytes were fixed in 1% paraformaldehyde and 0.2% glutaraldehyde in HEPES-buffered saline (pH 7.05) and embedded in LR White resin (Polysciences, Warrington, PA, USA). Sections were blocked for 30 min in PBS containing 5% skim milk and 0.01% Tween 20 (PBS-milk-Tween 20), incubated overnight at 4 °C in PBS-milk-Tween 20 containing either rabbit anti-PyCryPH2 antiserum (1:250 dilution) or rabbit anti-PyCryPH antiserum (1:50 dilution), and then incubated at 37 °C for 1 h in PBS-milk-Tween 20 containing goat anti-rabbit IgG conjugated with 15 nm gold particles (BBI Solutions, Crumlin, UK) diluted 1:40 in PBS-milk-Tween 20. The sections were stained with 2% uranyl acetate in 50% methanol, followed by Reynold’s lead citrate solution, and then examined by transmission electron microscope (JEM-1230; JEOL, Tokyo, Japan).

### 2.6 Generation of transgenic parasites

The *pycryph2* and *pycryph* genes are situated in a head-to-head orientation, and were simultaneously replaced by double crossover homologous recombination, using a drug selectable marker which encodes a fusion protein of human dihydrofolate reductase (hDHFR) and yeast cytosine deaminase and uridyl phosphoribosyl transferase (yFCU) (Lin et al., 2011). The resulting double disruptant parasite line was named Δ[CryPH/CryPH2]. Taking advantage of the head-to-head genomic arrangement, the disruption plasmid was constructed to include the 3′ flanking regions of both genes in the corresponding orientations upstream and downstream of the drug selection marker expressing cassette in the plasmid pL0048 (kindly provided by Dr. Chris J. Janse and Dr. Takeshi Annoura). The 3′ flanking region (730 bp) of *pycryph* and the 3′ flanking region of (802 bp) of the *pycryph2* were amplified from *P. yoelii* 17XNL genomic DNA (gDNA) using PyCryPH-3-rev-F-HindIII (5′‐gctatgcatcaagcttTAATTTTATTCATATCA‐3′) and PyCryPH-3-rev-R-KpnI (5′‐gtggatccgagctcggtaccGAATAGGGAAAAAAAAATCTCC‐3′), PyCryPH2-3-F-EcoRV (5′‐gcgaattctgcagatatcGACCAAAAGGGAAACAAAAAA‐3′) and PyCryPH2-3-R‐ApaI (5′‐ctatagggcgaattgggcccCGAAAAATGGATATGTGCCA‐3′), respectively. The fragments were inserted into HindIII and KpnI sites or EcoRV and ApaI sites, respectively, of the plasmid pL0048, which was then digested with HindIII and ApaI before transfection. Enriched schizonts of *P. yoelii* 17XNL were transfected with 20 μg of digested plasmid by electroporation using Nucleofector (Lonza Japan Ltd., Tokyo, Japan) with a human T cell Nucleofector kit solution and the U‐33 program, and then parasites were selected with pyrimethamine (Janse et al., 2006).

*pycryph2* disrupted parasites (ΔCryPH2) were generated by replacing the drug selectable marker cassette in the Δ[CryPH/CryPH2] line with a wild type *pycryph* gene. This genetic restoration allowed for phenotypic analysis of *pycryph2* disruption. To construct the transfection plasmid, the hDHFR::yFCU drug selectable marker cassette region in the above pL0048 plasmid was replaced by the full-length PyCryPH coding sequence along with its 5’ UTR, which was amplified from *P. yoelii* 17XNL gDNA using PyCryPH-F (5’-tttttttccctattcGTTTTAACTATGTATGTGCATT-3’) and PyCryPH-R (5’-tgtttcccttttggtagTTGTGTGCAAGTGTGTAT-3’). Plasmid digestion and DNA transfection to Δ[CryPH/CryPH2] parasites were performed as described above, but using a Basic Parasite Nucleofector kit-2. DNA integrated parasites at the expected locus would result in the loss of the negative selectable marker, yFCU, and were selected by treatment with 5-fluorocytosine (1.5 mg/ml; Sigma-Aldrich) in the mouse drinking water. The integration of the target DNA fragment was determined by genotyping PCR. Each transgenic parasite was cloned by limiting dilution.

Control parasites (Comp[CryPH/CryPH2]) were generated by replacing the drug selectable marker cassette in Δ[CryPH/CryPH2] parasite with both of the native *pycryph* and *pycryph2* genes. To generate a replacement construct for *pycryph*/*pycryph2*, the hdhfr::yfcu selectable marker cassette in the pL0048 plasmid constructed described above was replaced with full length *pycryph* and *pycryph2*, including each 5’ UTR amplified from *P. yoelii* 17XNL gDNA using PyCryPH-F and PyCryPH2-R (5’-tgtttcccttttggtCTATAAATTATCATCATCATTATC-3’). DNA transfection to Δ[CryPH/CryPH2] parasites and cloning of control parasites (Comp[CryPH/CryPH2]) were performed as described above.

### 2.7 PCR genotyping

The DNA integration at the expected locus was confirmed by PCR genotyping using transgenic parasite genomic DNA as template and KOD FX polymerase (TOYOBO, Osaka, Japan) with the following conditions: 94 °C for 2 min, 30 cycles of 98 °C for 10 s, 55 °C for 30 s, 68 °C for 2 min, and 68 °C for 3 min. The endogenous and restored *pycryph2* loci were detected by PCR using primers, PH2-F2 (5’-TGTAATATCTCGAAAACCGGA-3’) and PH2-3-R2 (5′‐TATAATATCGTCCTTTCCCCTT‐3′). Endogenous and restored *pycryph* loci were detected by PCR using primers, PH-3-F1 (5′‐GAATTTGATGCTTTTCTATGAGG‐3′) and PH-R1 (5′‐CCGATATACCAAAGCATTCAAC-3’). The integration of the hdhfr::yfcu selectable marker cassette was detected by PCR using primers yFCU-F3 (5′‐GTGCCCTCGACAGAGGTCTAG‐3′) and PH2-3-R2. The PCR products were electrophoretically separated on 1.5% agarose gels.

### 2.8 Sporozoite infectivity to mice

*P. yoelii* 17XNL wild type or ΔCryPH2 sporozoites were collected from infected mosquito hemocoel at Day 13 post-feeding by infusion of 100 µl of RPMI 1640 medium. Five thousand sporozoites were intravenously inoculated into 5-week-old female BALB/c mice (n=6). Parasitemias of each mouse were assayed by microscopic examination of Giemsa stained thin-blood smears.

### 2.9 Sporozoite gliding motility assay

*P. yoelii* 17XNL wild type, ΔCryPH2, and Comp[CryPH/CryPH2] sporozoites were collected from mosquito hemolymph at days 13 post-feeding. For gliding assays, sporozoites were suspended in RPMI 1640 containing 60% FCS and were mixed with two volumes of Matrigel (Corning Inc., Corning, NY, USA), resulting in a final FCS concentration of 20%. The mixture was placed in glass-bottom dishes and incubated at 37 °C for 5 min. Sporozoite migration was recorded using an Axio Observer Z1 (Carl Zeiss) microscope every 2.6 seconds for up to 150 frames (z-stack slices were imaged at an interval of 3 µm for 8 slices). Experiments were repeated five times with at least 80 sporozoites per parasite line. Sporozoites were classified according to circulating, meandering, or non-motile gliding patterns (Nozaki et al., 2020), and the numbers within each category were counted using Fiji software (Schindelin et al., 2012).

### 2.10 RNA isolation, RNA-seq library preparation, and sequencing

Total RNA was extracted from oocyst-derived and hemolymph sporozoites at day 13 post-feeding on mice infected with ΔCryPH2 or Comp[CryPH/CryPH2] parasite lines. Oocyst-derived sporozoites were isolated from infected mosquito midguts and were purified by density gradient centrifugation using a 17% Accudenz solution (Accurate Chemical & Scientific Corporation). Hemolymph sporozoites were collected by perfusing RPMI 1640 medium through the mosquito thorax. Total RNA from the purified sporozoites was isolated using TRIzol reagent (Invitrogen, Thermo Fisher Scientific) according to the manufacturer’s instructions. RNA-seq libraries were prepared from the total RNA using an NEBNext Ultra II Directional RNA Library Prep Kit for Illumina (New England Biolab, Ipswich, MA, USA) and 150-bp paired-end reads with approximately 20 million reads per sample were sequenced on an Illumina NovaSeq platform. For oocyst-derived sporozoites, three independent biological replicates were performed for each parasite line. Raw sequencing data have been deposited in the DDBJ Sequence Read Archive (DRA) under accession number PRJDB35932.

### 2.11 Analysis of RNA-seq data

The obtained sequence data were mapped to the *P. yoelii* 17XNL reference genome from PlasmoDB-62 using HISAT2 (version 2.2.1), with the maximum intron length parameter set to 2000. To accurately quantify gene expression, a cDNA-only GFF file was generated from the *P. yoelii* 17XNL GTF file from PlasmoDB-62. Read counts for each gene were subsequently quantified from the mapped data using featureCounts (version 2.0.1) from the Subread package. Differential gene expression analysis of oocyst-derived sporozoite RNA-seq data was performed using the EdgeR R package (version 3.40.2). A generalized linear model (GLM) framework with a negative binomial distribution was employed to account for biological replicates, and the resulting p-values were adjusted for multiple testing using the Benjamini-Hochberg method to control for the false discovery rate (FDR). Genes were considered to be differentially expressed (DEGs), if their adjusted p-values (FDR) were less than 0.05. Unless otherwise specified, all program parameters were set to their default values. Gene Ontology (GO) enrichment analysis was conducted on DEGs from oocyst-derived sporozoites of ΔCryPH2 using the clusterProfiler R package (version 4.8.1). Separately, gene sets that were up-regulated (with a *log*_2_ fold change greater than 0.5) and down-regulated (with a *log*_2_ fold change less than -0.5) in ΔCryPH2 were analyzed. A custom GO-term database, created from the *P. yoelii* 17XNL GO annotation file from PlasmoDB-62, was utilized. GO terms with an FDR less than 0.05 were considered significantly enriched using the Benjamini-Hochberg method.

To identify common or unique gene expression patterns, previously reported RNA-seq data of *P. yoelii* 17XNL oocyst-derived sporozoites (n=3) and salivary glands-derived sporozoites (n=3) from Lindner et al. (2019) were re-analyzed using the pipeline described above to identify DEGs. For this comparative analysis, genes with a *log*_2_ fold change greater than 1 were defined as up-regulated in oocyst-derived sporozoites, while those with a *log*_2_ fold change less than -1 were defined as up-regulated in salivary glands-derived sporozoites (implying up-regulation in salivary glands-derived sporozoites relative to oocyst-derived sporozoites). Venn diagram analysis was performed using the VennDiagram package in R (version 1.7.3) to compare the up-regulated genes in oocyst-derived sporozoites of ΔCryPH2 with the DEGs recalculated from the previously reported data.

Principal component analysis (PCA) was performed using Rstats on the Transcripts Per Million (TPM) normalized read counts. TPM values were calculated for all samples to ensure comparability across different sequencing depths. The dataset for PCA included oocyst-derived sporozoites of ΔCryPH2 and Comp[CryPH/CryPH2] (n=3 for each), hemolymph sporozoites of Comp[CryPH/CryPH2] (n=1), and the re-analyzed RNA-seq data from Lindner et al. (2019). The first two principal components (PC1 and PC2) were visualized using plotPCA to illustrate the overall similarities and differences among the samples.

### 2.12 Statistical analysis

All statistical analyses were performed using Prism 9 software (GraphPad Software, Boston, MA, USA). Non-parametric Kruskal–Wallis tests followed by Dunn’s multiple comparison post-test were used to compare three groups (PyWT, ΔCryPH2, and Comp[CryPH/CryPH2]). Mann Whitney test was applied for comparison of parasitemias between parasite lines. Statistical significance was set at *P* < 0.05. Two asterisks (**) indicate P values less than 0.01.

## 3 Results

### 3.1 CryPH2 is localized to the crystalloid of ookinetes

**Figure 1:**
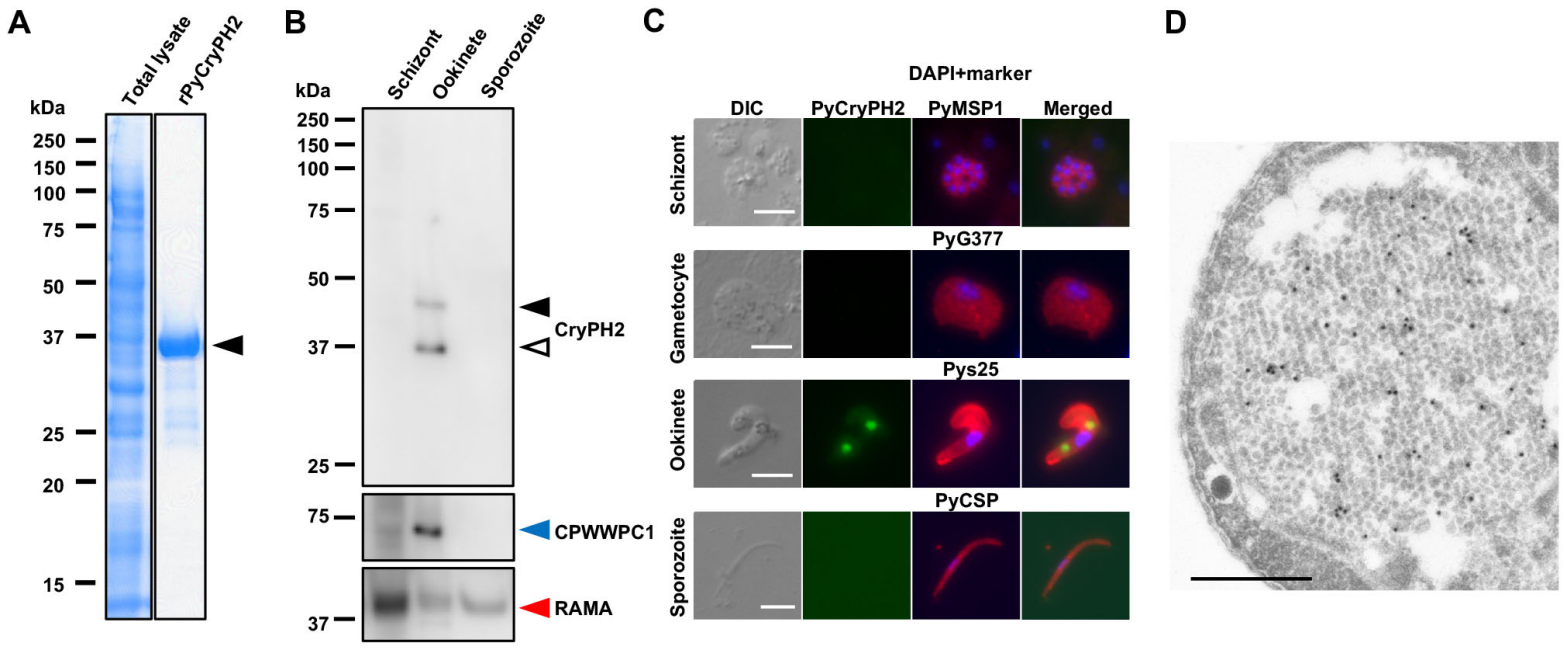
PyCryPH2 is localized to the crystalloids in *Plasmodium yoelii* ookinetes. **(A)** Expression of recombinant PyCryPH2 (aa 289 to 386) fused with a GST-tag at its N-terminus using the wheat germ cell-free protein expression system. Total lysate and recombinant PyCryPH2 purified using a glutathione Sepharose affinity column were separated using 12.5% SDS-polyacrylamide gel electrophoresis (SDS-PAGE). The major band, indicated by an arrowhead, corresponds to the recombinant PyCryPH2 fused with GST, whose calculated molecular weight is 37 kDa. **(B)** Western blotting analysis of PyCryPH2 in invasive stages. Protein lysates of schizonts, ookinetes, and salivary gland-derived sporozoites (1 × 10^5^) were separated by SDS-PAGE under a reducing condition. PyCryPH2 expression, indicated by arrowheads (closed: full length, open: processed form), was detected using rabbit anti-PyCryPH2 antiserum. Ookinete formation was confirmed by antiserum against the ookinete marker PyCPWWPC-1 (Kangwanrangsan et al., 2013, middle panel, indicated by a blue arrowhead). Protein loading was assessed by re-probing with anti-PbRAMA antiserum (lower panel, red arrowhead). **(C)** Localization analysis of PyCryPH2 by immunofluorescence assay. Schizonts, gametocytes, ookinetes, and sporozoites of *P. yoelii* 17XNL were fixed with 4% paraformaldehyde and permeabilized with 0.1% Triton X-100 treatment, followed by incubation with rabbit anti-PyCryPH2 antiserum (shown in green). Antisera against PyMSP1, PyG377, Pys25, and PyCSP were used as specific markers (red) for schizonts, gametocytes, ookinetes, and sporozoites, respectively. Nuclei were stained with DAPI (blue). DIC, differential interference contrast microscopy image. Bar, 5 µm. **(D)** Localization analysis of PyCryPH2 by immunoelectron microscopy. Gold particles corresponding to PyCryPH2 were observed in the crystalloid in the cytoplasm of an ookinete. Bar, 500 nm.

To determine PyCryPH2 localization, specific rabbit antibodies were raised against recombinant protein corresponding to the C-terminus adjacent to the PH domain of PyCryPH2 (289 aa to 386 aa), a region which has limited similarity to PyCryPH. The recombinant protein was fused at its N-terminus to glutathione S-transferase and was produced using the wheat germ protein expression system (**Figure 1A**). The specificity of the antisera collected from an immunized rabbit was determined by western blotting using lysates from schizont, ookinete, or sporozoite stages of *Plasmodium yoelii* 17XNL. Ookinetes were prepared by *in vitro* culture of parasite infected erythrocytes and confirmed by antibodies against the ookinete specific protein, CPWWPC-1. Western blot assays detected two proteins in ookinete lysates at approximately 44 kDa and 37 kDa (**Figure 1B**), which might correspond to full-length and processed forms of PyCryPH2. PyCryPH2 is localized specifically to crystalloids in ookinetes, as determined by indirect immunofluorescent assay using the specific antibodies (**Figure 1C**). Immunoelectron microscopic analysis further confirmed that PyCryPH2 is exclusively localized to crystalloid particles shown as a honeycomb structure (**Figure 1D**, **Supplementary Figure 1**), similar to PyCryPH (Jenwithisuk et al., 2018).

### 3.2 Generation of *cryph2* gene disrupted parasites

**Figure 2:**
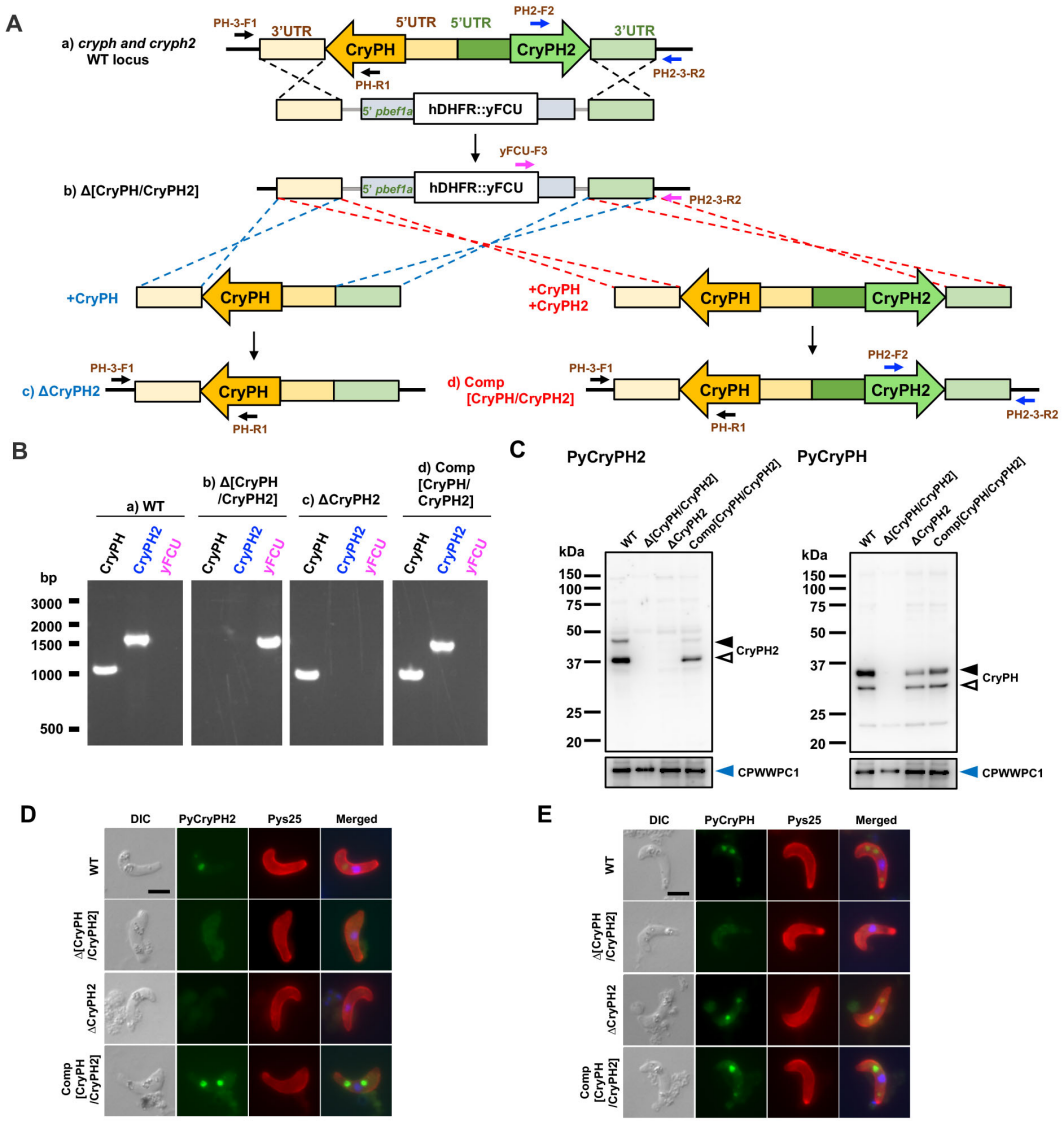
Generation of *pycryph2*-disrupted parasites and control parasites. **(A)** Schematic representation of the generation of *pycryph/pycryph2* (upper) and *pycryph2* (lower left) targeted gene disruption, together with control parasites which were restored for both *pycryph* and *pycryph2* (lower right). *Pycryph/pycryph2* double knockout parasites (Δ[CryPH/CryPH2]) were generated by replacing the *pycryph* and *pycryph2* loci with an expression cassette of hDHFR (human dihydrofolate reductase: positive selectable marker) and yFCU (yeast cytosine deaminase and uridyl phosphoribosyl transferase: negative selectable marker) as a fusion protein (hDHFR::yFCU) by double-crossover homologous recombination. Δ[CryPH/CryPH2] transgenic parasites were selected by positive selection with pyrimethamine. *Pycryph2*-disrupted parasites (ΔCryPH2) and control parasites (Comp[CryPH/CryPH2]) were generated by replacing the hDHFR::yFCU expression cassette in Δ[CryPH/CryPH2] parasites with *pycryph* gene or both *pycryph* and *pycryph2* genes by double-crossover homologous recombination, respectively. Both transgenic parasites were selected by negative selection with 5-fluorocytosine treatment. Locations of primers used for genotyping PCR analyses are shown as arrows. **(B)** Genotyping PCR to confirm DNA integration in the expected region in the transgenic parasite genome. PCRs were performed using the primers as shown in **(A)** to detect *pycryph*, *pycryph2*, or *hdhfr::yfcu*, from genomic DNA extracted from each transgenic parasite line. DNA amplification using a primer set of PH-3-F1 and PH-R1 (1055 bp), or PH2-F2 and PH2-3-R2 (1552 bp) demonstrated that *pycryph* or *pycryph2* loci remained, respectively. In contrast, DNA amplification using a primer set of yFCU-F3 and PH2-3-R2 (1554 bp) demonstrated that the locus for the two genes was replaced by a *hdhfr::yfcu* expressing cassette. Panels of electrophoresis show the result of PCR genotyping using parasite genomic DNA indicated at the top. We confirmed that only the *pycryph2* locus was deleted in ΔCryPH2 parasites and that both genes were restored in control (Comp[CryPH/CryPH2]) parasites. **(C)** Detection of PyCryPH and PyCryPH2 expression in wild type (WT) and transgenic parasites. Protein lysates of cultured ookinetes (1 × 10^5^) of each parasite line indicated above were separated by SDS-PAGE under a reducing condition. PyCryPH2 (left panels) or PyCryPH (right panels) expression was detected using specific antibodies. As shown in **Figure 1B**, two bands, corresponding to the full-length and processed forms of PyCryPH2 were detected in ookinete lysates of wild type and control (Comp[CryPH/CryPH2]). Using anti-PyCryPH antiserum, two bands appeared, corresponding to full-length at approximately the calculated size (33.7 kDa) and a processed form as reported (Jenwithisuk et al., 2018). In ΔCryPH2 ookinetes, only PyCryPH was detected by antibodies, confirming that *pycryph2* alone disrupted parasites were obtained. In control ookinetes, both PyCryPH and PyCryPH2 were detected as in wild type. Anti-PyCPWWPC1 antiserum was used as the loading control (lower panels). **(D, E)** Detection of PyCryPH **(D)** and PyCryPH2 **(E)** localization in ookinetes of transgenic parasites. The cultured ookinete of each parasite line was fixed in 4% paraformaldehyde and permeabilized with 0.1% Triton X-100. The ookinetes were incubated with anti-PyCryPH **(D)** or anti-PyCryPH2 **(E)** antiserum (shown in green). Antiserum against Pys25 was used as a specific marker for the ookinete surface (shown in red). Nuclei were stained with DAPI (shown in blue in the merged images). Only PyCryPH and not PyCryPH2 was detected in crystalloids in ΔCryPH2 ookinetes, further confirming the successful generation of *pycryph2*-disrupted parasites. Bar, 5 µm.

To investigate the role of PyCryPH2 in mosquito stages, we generated *pycryph2* gene disrupted parasites (ΔCryPH2). Initially, we attempted to obtain ΔCryPH2 transgenic parasites by homologous recombination to replace the endogenous *pycryph2* locus in the *P. yoelii* 17XNL genome with an hDHFR and yFCU expression cassette as a selectable marker. We also planned to generate *pycryph* and *pycryph2* double disrupted parasites (Δ[CryPH/CryPH2]) by homologous recombination as above, since these genes are located adjacent in the genome (**Figure 2A**). Several attempts did not succeed to isolate ΔCryPH2 parasites, while Δ[CryPH/CryPH2] transgenic parasites were generated. Therefore, we next attempted to complement Δ[CryPH/CryPH2] transgenic parasites with PyCryPH, by replacement with an hDHFR::yFCU cassette, to obtain *pycryph2* disrupted parasites (ΔCryPH2). In addition, by simultaneously complementing Δ[CryPH/CryPH2] with a PyCryPH and PyCryPH2 expressing cassette, we generated control parasites (Comp[CryPH/CryPH2]) equivalent to wild type.

Gene disruption and complementation were confirmed by genotyping PCR (**Figure 2B**). Western blotting using specific antibodies against PyCryPH or PyCryPH2 showed that neither PyCryPH nor PyCryPH2 were detected in Δ[CryPH/CryPH2] ookinete lysates, while PyCryPH was restored in ΔCryPH2 ookinetes. Following replacement of both genes, PyCryPH and PyCryPH2 proteins were detected in Comp[CryPH/CryPH2] ookinetes similar to wild type (WT) (**Figure 2C**). Immunofluorescent analyses revealed that only PyCryPH2 was absent in crystalloids of ΔCryPH2 ookinetes, while both were undetectable in Δ[CryPH/CryPH2] ookinetes (**Figures 2D, E**). From these results, we concluded that *pycryph2* gene disruption parasites were successfully generated from Δ[CryPH/CryPH2] transgenic parasites, with suitable control parasites expressing both PyCryPH and PyCryPH2 similar to wild type.

### 3.3 Characterization of crystalloid formation in *cryph2* disrupted parasites

**Figure 3:**
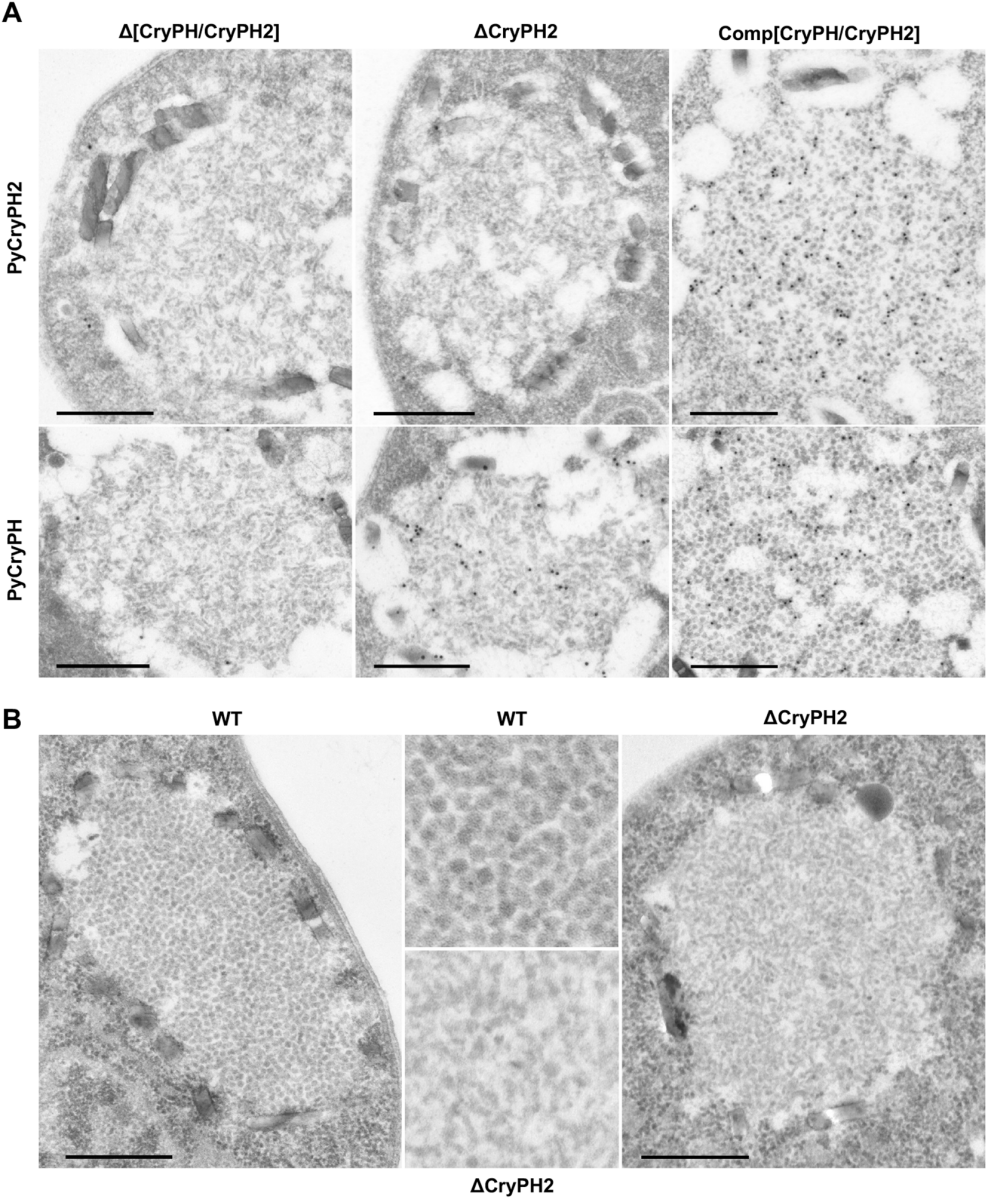
PyCryPH2 is involved in microstructure formation of the crystalloid in ookinetes. **(A)** Immuno-EM was performed to investigate crystalloid formation and PyCryPH2 and PyCryPH localization in ookinetes of transgenic parasites. All images show crystalloids in the ookinete of transgenic parasites. In the ookinete cytosol, crystalloids, defined as honey-comb-like structures surrounded by hemozoin crystals, were detected in all examined transgenic parasite lines. Gold particles showing the localization of PyCryPH2 (upper panels) or PyCryPH (lower panels) demonstrate that PyCryPH2 was eliminated while PyCryPH was restored in crystalloids of ΔCryPH2 ookinetes. In control (Comp[CryPH/CryPH2]) ookinetes, it was confirmed that both PyCryPH and PyCryPH2 were restored in crystalloids, as in wild type. Scale bar, 500 nm. **(B)** Conventional EM images to investigate the detailed morphology of the particles within the crystalloids. Subunit-like particles in the crystalloid are observed in wild type and ΔCryPH2 ookinetes. In wild type (left), the crystalloid subunit particles have a characteristic spherical shape, whereas in ΔCryPH2 ookinete (right), the particles are somewhat club-shaped. The images in the center correspond to magnified views of wild type (upper) and ΔCryPH2 (bottom). The scale bar indicates 500 nm.

Ookinete crystalloids contain numerous internal particles which are often observed as a crystalline-like array. Morphological analyses of mature ookinetes by electron microscopy revealed that crystalloids, surrounded by hemozoin crystals, developed in Δ[CryPH/CryPH2] and ΔCryPH2 parasites as well as in the control (**Figure 3A**). Unlike for the LAP null mutant (Carter et al., 2008; Saeed et al., 2015), crystalloids were observed in ΔCryPH2 parasites. Gold particles indicating the reaction of antibodies against PyCryPH were detected inside the crystalloid of ΔCryPH2, whereas in control parasites it was detected by both PyCryPH and PyCryPH2 antibodies, which was consistent with the results of immunofluorescent analyses. Higher magnification images demonstrated that crystalloids are filled with spherical particles in an orderly manner in wild type ookinetes. In contrast, in ΔCryPH2 crystalloids, the particles were oval or club-shaped and were irregularly packed (**Figures 3A, B**). These results indicate that PyCryPH2 is required for the formation of clusters of subunit particles inside the crystalloids.

### 3.4 *cryph2* disrupted parasites produce sporozoites but fail to invade salivary glands

**Figure 4:**
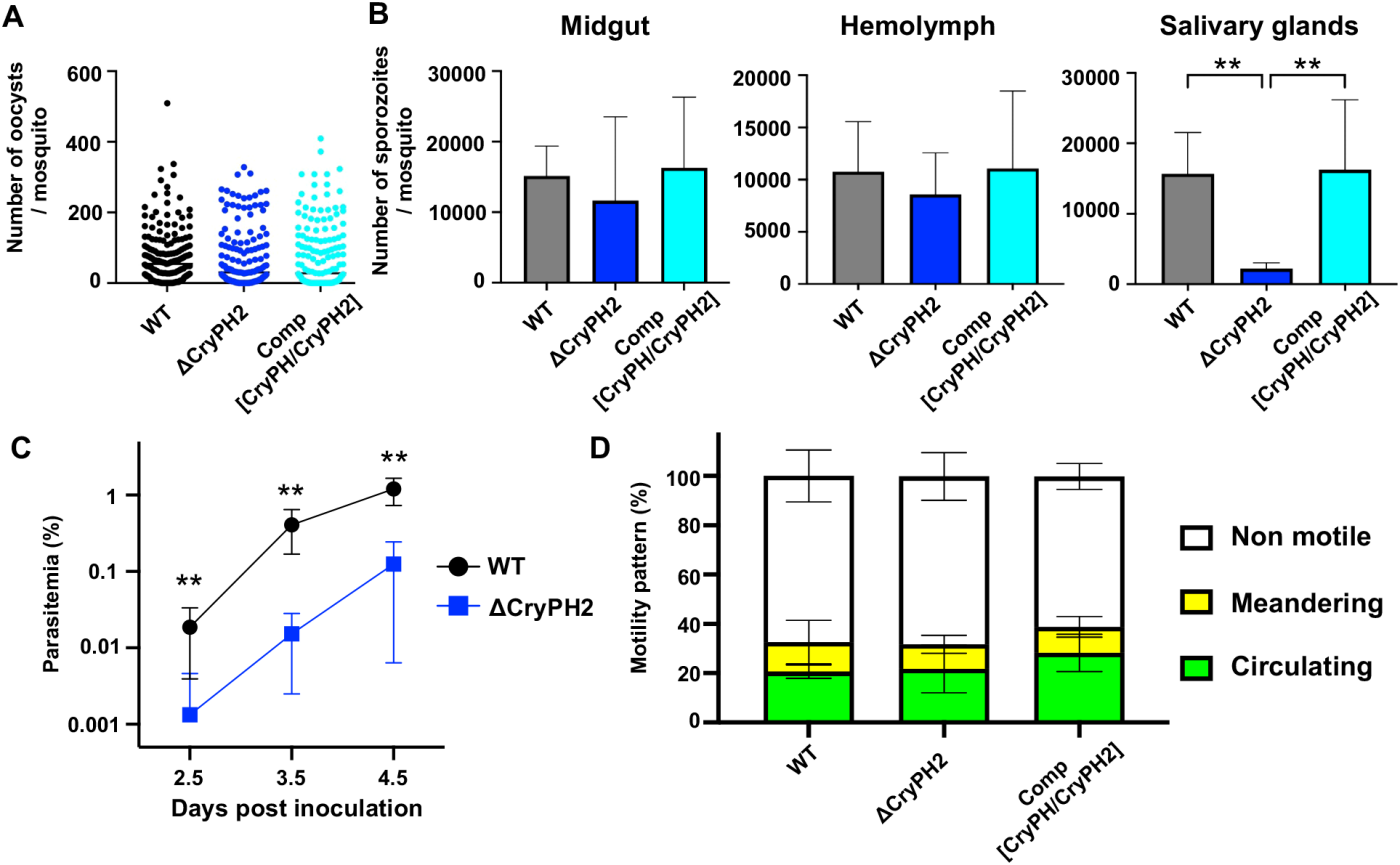
*pycryph2*-disruption impaired the ability of sporozoites to invade salivary glands. **(A)** Numbers of mosquito midgut oocysts were counted at day 8 post-feeding from 8 independent experiments. **(B)** The numbers of sporozoites collected from midguts (left), hemolymph (middle), and salivary glands (right) at days 12, 13 and 14 post-feeding (n=5, 6, 8 for each), respectively, were compared among wild type, ΔCryPH2, and control (Comp[CryPH/CryPH2]) parasites. Midgut oocyst numbers were not significantly different among these parasite lines. Sporozoite formation and their release into hemocoel were similar among all lines, whereas sporozoites collected from salivary glands were significantly reduced in the ΔCryPH2 line; analyzed by the Kruskal-Wallis test with a Dunn’s *post hoc* test (**P < 0.01). **(C)** Sporozoite infectivity for mice. Hemolymph sporozoites were harvested from WT- or ΔCryPH2-infected mosquitoes at day 13 post-feeding. Five-thousand sporozoites were inoculated intravenously into BALB/c mice (n=5). Average parasitemias were determined daily by Giemsa-stained blood smears, and are plotted with standard deviations as error bars. Parasitemias of each day in ΔCryPH2 sporozoite inoculated mice were significantly lower than those of the control (Mann Whitney test, **P < 0.01). **(D)** Sporozoite motility was compared among wild type (WT) and transgenic parasite lines. The gliding motility of sporozoites collected from hemolymph at day 13 post-feeding was examined *in vitro*. Sporozoites were embedded in Matrigel and their movements were captured for 6 min by time-lapse imaging with an inverted microscope. Gliding patterns were classified into circulating (green), meandering (yellow), and non-motile (white), according to Nozaki et al. (2020). The stacked bar chart shows the percentages of sporozoites showing each motility pattern with standard deviations from five independent experiments with at least 80 sporozoites per parasite line. No significant difference was observed in the gliding motility of ΔCryPH2 sporozoites compared to wild type and control by statistical analysis using the Kruskal-Wallis.

Next, we investigated the effects of *pycryph2*-disruption on sporozoite formation and their capacity for invasion. The numbers of midgut oocysts at day 8 post-feeding of ΔCryPH2 infected mice (mean: 68 ± 7) were comparable to those of wild type (mean: 72 ± 6) and control parasites (mean: 75 ± 7) (**Figure 4A**). To determine the efficiency of sporozoite formation, egress, and invasion of salivary glands, sporozoites numbers collected from midguts (12 days), hemolymph (13 days), or salivary glands (14 days) were compared with wild type, control (Comp[CryPH/CryPH2]), and ΔCryPH2 parasites. Sporozoite numbers of ΔCryPH2 collected from midguts and hemolymph were not significantly different from those of wild type and control parasites; however, sporozoite numbers of ΔCryPH2 in salivary glands were reduced by approximately six-fold compared to wild type or control parasites (**Figure 4B**). These results suggest that disruption of *pycryph2*, which leads to the disordered formation of the crystalloid microstructure, impairs the ability of sporozoites to invade salivary glands, despite their morphologically normal formation and efficient release into the hemocoel. The double disrupted parasites, Δ[CryPH/CryPH2], exhibited a phenotype consistent with that of the *pycryph2* single disrupted parasites; and showed normal oocyst and sporozoite formation, but had a significantly reduced ability to invade salivary glands (**Supplementary Figure 2**). These findings reinforce the conclusion that the observed defect is specifically attributable to *pycryph2* disruption; particularly considering that PyCryPH has been shown to be dispensable throughout the life cycle (Jenwithisuk et al., 2018).

To examine whether the low number of salivary gland-residing ΔCryPH2 sporozoites was due to delayed maturation, sporozoites were collected from midguts and salivary glands longitudinally on days 12, 14, and 16 post-feeding. In all parasite lines, wild type, ΔCryPH2, and Δ[CryPH/CryPH2], midgut sporozoite numbers decreased in a time-dependent manner, indicating that sporozoite egress occurs similarly across these lines (**Supplementary Figure 3A**). As previously reported (Zhang et al., 2016), *P. yoelii* 17XNL sporozoites in salivary glands increased towards day 14 post-feeding, followed by a drastic reduction. This indicates that egressed sporozoites rapidly invade salivary glands but are not retained there for an extended period. In contrast, the numbers of *pycryph2* disrupted sporozoites remained consistently low, even as midgut sporozoite numbers decreased (**Supplementary Figure 3B**). These findings indicate that *pycryph2* disruption impaired the sporozoite invasion ability, rather than delaying their maturation.

Furthermore, ΔCryPH2 sporozoite infectivity for mice was examined by intravenous inoculation of hemolymph sporozoites into female BALB/c mice. Their parasitemias, compared to those of mice inoculated with control sporozoites, are shown in **Figure 4C**, demonstrating that *pycryph2* disruption also decreased sporozoite infectivity to mammalian livers by approximately 25-fold compared to the control.

Since ΔCryPH2 sporozoites showed decreased abilities to invade mosquito salivary glands as well as mouse livers, we further investigated sporozoite motility, required for both invasion processes and depends on the secretion of micronemal and rhoptry proteins from the sporozoite apical complex (Sultan et al., 1997; Ishino et al., 2019; Braumann et al., 2023). Sporozoites were collected from the hemolymph of parasite infected mosquitoes at day 13 post-feeding and were embedded in Matrigel to observe their movement under a microscope. The movement patterns of sporozoites were categorized as circulating, meandering, and non-motile (Nozaki et al., 2020); and their percentages were shown as bar graphs (**Figure 4D**). No significant difference in sporozoite motility was detected between ΔCryPH2 and control parasites, suggesting that PyCryPH2 is not crucial for sporozoite motility.

### 3.5 Transcriptome analysis reveals a functionally immature state of Δ*CryPH2* sporozoites

**Figure 5:**
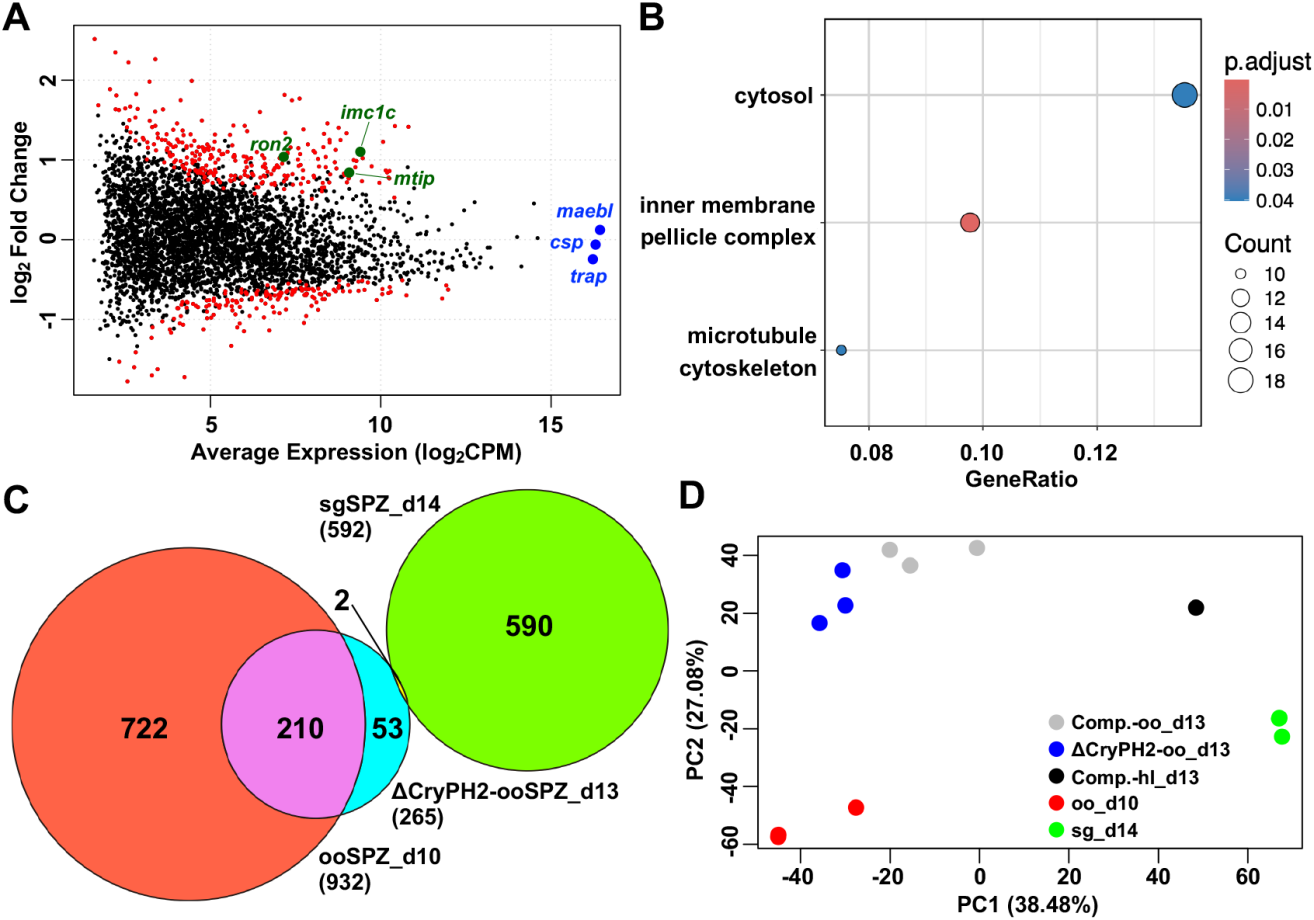
Developmental arrest of ΔCryPH2 sporozoites revealed by transcriptomic analysis. **(A)** MA plot of differentially expressed genes (DEGs) in *P. yoelii* oocyst-derived sporozoites. The MA plot visualizes the average log Counts Per Million (CPM) (x-axis) versus the logFC (y-axis) for all genes in ΔCryPH2 versus complemented parasite oocyst-derived sporozoites. Red points represent genes identified as significantly differentially expressed (FDR < 0.05). Representative up-regulated genes (*imc1c*, *mtip*, and *ron2*) are highlighted in green, while representative genes with no significant change in expression (*csp*, *trap*, and *maebl*) are highlighted in blue. The source data is **Supplementary Table 2**. **(B)** Gene Ontology (GO) enrichment analysis of differentially expressed genes (DEGs) in ΔCryPH2 oocyst-derived sporozoites. Bubble plots display enriched GO terms (FDR < 0.05) for genes that were up-regulated (log_2_ fold change > 0.5) or down-regulated (log_2_ fold change < -0.5) in ΔCryPH2 oocyst-derived sporozoites compared to control (Comp[CryPH/CryPH2]) parasites. The x-axis represents the gene ratio, indicating the proportion of differentially expressed genes (DEGs) relative to the total number of genes in each GO term. The dot size corresponds to the number of DEGs (count), and the color gradient represents the statistical significance based on the adjusted p-value. **(C)** Venn diagram analysis of shared and unique DEGs. A Venn diagram illustrates the overlap between genes up-regulated in ΔCryPH2 oocyst-derived sporozoites (ooSPZ) (log_2_ fold change > 0.5) and DEGs from previously reported RNA-seq data (Lindner et al., Nat Commun. 2019) showing up-regulation in oocyst-derived sporozoites (log_2_ fold change > 1) or salivary glands-derived sporozoites (sgSPZ) (log_2_ fold change < -1). Numbers indicate the count of genes in each section. **(D)** Principal Component Analysis (PCA) of *P. yoelii* sporozoite RNA-seq data. A 2D PCA plot generated from TPM-normalized read counts, showing the relationships among oocyst-derived sporozoites (oo) of ΔCryPH2 and control (Comp[CryPH/CryPH2]) parasites (n=3 each), hemolymph sporozoites (hl) of control (Comp[CryPH/CryPH2]) parasites (n=1), and re-analyzed published RNA-seq data from Lindner et al. (2019) (oocyst-derived (oo) and salivary glands-derived (sg), n=3 each). PC1 and PC2 explain the indicated percentages of total variance. Each point represents a single sample.

To investigate the reduced invasion ability due to *pycryph2* disruption, transcriptome analysis was performed by RNA-seq using oocyst-derived sporozoites of ΔCryPH2 and control parasites (Comp[CryPH/CryPH2]). Approximately 2 to 6 million reads from the oocyst-derived sporozoite data were mapped to the *P. yoelii* genome (**Supplementary Table 1**). The number of mapped reads exceeds that reported in a previous RNA-seq study of *P. yoelii* sporozoites (Lindner et al., 2019), indicating that the read depth was sufficient for the analysis. Using the RNA-seq data, we performed a differential expressed gene (DEG) analysis between ΔCryPH2 and the complemented oocyst-derived sporozoites. An MA-plot was generated to visualize the data, and compared the average expression of all genes against their fold change (**Figure 5A**, **Supplementary Table 2**). **Supplementary Tables 3** and **4** list genes with a significant expression change of at least 0.5 log_2_ fold change in either direction (≥ 1.41-fold upregulation or ≥ 1.41-fold downregulation). The mRNA levels of known motility- or invasion-related micronemal proteins, such as thrombospondin-related anonymous protein (TRAP) and membrane associated erythrocyte binding-like protein (MAEBL), as well as circumsporozoite protein (CSP), were not significantly decreased by *pycryph2* disruption, which corresponds well to their observed normal gliding motility (**Figure 5A**, **Supplementary Figure 3**). Gene Ontology (GO) analysis revealed no significantly enriched categories among the down-regulated genes in ΔCryPH2 sporozoites.

The transcription of some known genes related to the inner membrane complex, such as myosin A-tail interacting protein (MTIP), inner membrane complex protein 1c (IMC1c)/alveolin 5 (ALV5), and rhoptry neck protein 2 (RON2) were enhanced in ΔCryPH2 sporozoites (**Figure 5A**, **Supplementary Figure 3**). Therefore, we applied gene ontology analysis, resulting in significant enrichment of inner membrane pellicle complex group molecules, including both MTIP and IMC1c, in the genes upregulated by *pycryph2* disruption (**Figure 5B**). The inner membrane complex (IMC) is required for sporozoite elongated structure and motility and is formed at an earlier stage during sporogony; thus gene expression related to IMC is upregulated in oocyst-derived sporozoites compared to further matured sporozoites collected from salivary glands (Lindner et al., 2019). According to this finding, we next categorized all upregulated genes (>0.5 log_2_ in ΔCryPH2 sporozoites) depending on expression pattern in oocyst-derived sporozoites (at day 10 post-feeding, ooSPZ) and salivary gland sporozoites (at day 14 post-feeding, sgSPZ) using published RNA-seq dataset on *P. yoelii* 17XNL line sporozoites (Lindner et al., 2019). As shown in **Figure 5C**, most upregulated genes (210 out of 265 genes) were included in genes expressed higher in oocyst-derived sporozoites by more than 2-fold than those in salivary gland sporozoites. The findings that most genes showing higher expression levels in oocyst-derived sporozoites by *pycryph2* disruption are repressed according to sporozoite maturation in wild type raises the possibility that ΔCryPH2 sporozoites remain functionally immature inside oocysts. We then extended the expression pattern comparison to all genes using principal component analysis (PCA) (**Figure 5D**). The RNA-seq data for this analysis included ΔCryPH2 and control oocyst-derived purified sporozoites at day 13 post-feeding, control hemolymph sporozoites at day 13 post-feeding, as well as the publicly available dataset from Lindner et al. (2019), which contains oocyst-derived sporozoites at day 10 post-feeding and salivary gland sporozoites at day 14 post-feeding. The PC1 axis of the analysis appeared to reflect the process of sporozoite maturation. Specifically, oocyst-derived sporozoites at day 10 post-feeding were located to the left, while hemolymph sporozoites at day 13 and salivary gland sporozoites were positioned towards the right, indicating a progression along this axis. The ΔCryPH2 oocyst-derived sporozoites collected at day 13 were situated between the day 10 wild type sporozoites and day 13 control sporozoites, both of which were collected from oocysts.

This analysis provides further support for the hypothesis that the ΔCryPH2 sporozoites remain in an immature state inside oocysts, which may lead to their reduced ability to invade salivary glands. Further investigations are required to elucidate the specific mechanism by which the disordered formation of crystalloid microstructure, caused by the disruption of *pycryph2*, impacts the sporozoite functional maturation sufficient to invade salivary glands, particularly since both PyCryPH2 and crystalloids are absent in sporozoites.

## 4 Discussion

The pleckstrin homology (PH) domain consists of approximately 120 amino acids and is widely found in eukaryotes, and is known to be involved in intracellular signal transduction and interactions with membranes (Lemmon, 2007; Powis et al., 2023). In *Plasmodium*, several molecules containing a PH domain have been reported to function in the erythrocytic stage; including the kinase PfCDPK7 which is involved in the cell cycle, and molecules such as APH and RASP which are implicated in merozoite invasion (Kumar et al., 2014; Suarez et al., 2019; Chaiyawong et al., 2022). We previously reported that the PH domain protein PyCryPH (PY17X_0705200) specifically localizes to the crystalloids of *P. yoelii* ookinetes; however, no phenotypic changes were observed in *pycryph* disrupted parasites (Jenwithisuk et al., 2018). In this study, we focused on another PH domain containing protein, PyCryPH2, whose gene is adjacent and in a head-to-head orientation with respect to *pycryph* in the genome. By immuno electron microscopy we confirmed that native PyCryPH2 similarly localizes to ookinete crystalloids. Functional analysis was performed by generating *pycryph2* disrupted parasites, and in this parasite line crystalloids were observed under a light microscope. In the rodent malaria parasite *Plasmodium berghei* it was reported that the loss of the crystalloid proteins LAP1 or LAP3 completely inhibit the formation of ookinete crystalloids (Carter et al., 2008; Saeed et al., 2015). Moreover, it has been reported that GFP tagging of LAP4 leads to an abnormal crystalloid structure in ookinetes, characterized by clustered subunit vesicles. While this abnormality does not impair sporozoite formation within the oocysts, it affects subsequent processes, including sporozoite egress and their ability to invade salivary glands (Saeed et al., 2018). Description of *pycryph2*-deficient ookinetes by electron microscopy revealed that the particles within the crystalloid were not spherical, which is characteristic of wild type parasites, but rather were elongated-oval or club-shaped. While studies have reported a membrane-bound architecture for crystalloid subunits (Meis and Ponnudurai, 1987; Saeed et al., 2015), our current observations did not provide sufficient resolution to clearly identify these structures. Further investigations using higher-resolution techniques might elucidate the potential role of PyCryPH2 in maintaining the crystalloid microstructure. Thus, the loss of proteins localized to the crystalloids lead to phenotypes ranging in degree from anomalies in the fine internal structure to the complete disruption of crystalloids.

The relationship between the characteristic structure of the crystalloid and its roles in ookinetes, oocysts, and sporozoite development are questions to be addressed; and at this juncture are approached by describing phenotypic changes following targeted gene disruptions. The disruption of the ookinete crystalloid structure by PbLAP1 deletion resulted in the failure of sporogony in oocysts at the next stage (Claudianos et al., 2002; Carter et al., 2008). In addition, deletion of other LAP family members also resulted in severely impaired sporozoite formation within oocysts, regardless of whether or not crystalloid formation was affected (Claudianos et al., 2002; Raine et al., 2007; Carter et al., 2008; Ecker et al., 2008; Lavazec et al, 2009). Therefore, we investigated the effects of *pycryph2* disruption on sporozoite formation and maturation, and found that, unlike with PbLAP, *pycryph2* deletion did not impair sporozoite formation and release into the hemocoel; rather, the efficiency of salivary gland invasion as well as mouse liver infection was significantly decreased (**Figures 4B, C**). The phenotype is similar to that observed following disruption of the recently identified crystalloid protein CRYSP (Ukegbu et al., 2023). These results indicate that the loss of some crystalloid proteins affects sporozoite maturation indirectly, even though they are absent in sporozoites (**Figure 1C**). A time-course sporozoite collection assay demonstrated that both ΔCryPH2 and Δ[CryPH/CryPH2] sporozoites egressed from oocysts with similar timing to the wild type. However, the number of salivary gland-residing sporozoites remained lower throughout the observation period until day 16 post-feeding, at which point the number of wild type sporozoites collected from salivary glands had significantly reduced (**Supplementary Figure 3**). The results further indicate that the reduction in salivary gland sporozoite numbers by *pycryph2* disruption was not due to delayed maturation, but rather to functional perturbations during sporogony. The reduction in invasion ability cause by *pycryph2* disruption was further confirmed by the sporozoite infectivity assay in mice (**Figure 4C**). It is noteworthy that ΔCryPH2 sporozoites showed approximately 25-fold lower infectivity than wild type, a reduction comparable that seen in salivary gland invasion, although their motility was not significantly different from control sporozoites.

To better understand the relationship of gene expression patterns and the observed phenotype, transcriptomes were analyzed by RNA-seq to investigate any disorders occurring in developing sporozoites in oocysts caused by *pycryph2* disruption. RNA-seq results showed that key transcript levels of major sporozoite molecules implicated in sporozoite motility or invasion of salivary glands, such as, CSP, TRAP, and MAEBL (Ghosh and Jacobs-Lorena, 2009; Kojin and Adelman, 2019), in *pycryph2*-disrupted sporozoites were comparable to those in wild type sporozoites; which is consistent with the observation that *in vitro* motility of mutant sporozoites were not significantly impaired. In contrast, some gene groups were significantly upregulated in ΔCryPH2 sporozoites, especially those required for sporogony at their early stage, such as the inner membrane complex. By comparison of transcriptome data, it is notable that most upregulated genes in ΔCryPH2 sporozoites are highly expressed in oocyst-derived sporozoites and then repressed in sporozoites collected from salivary glands during wild type sporozoite maturation. This in silico analysis indicated that sporozoite maturation inside oocysts was arrested by *pycryph2* disruption, resulting in impaired invasion of salivary glands. The interruption was supported by the ΔCryPH2 sporozoite dynamics in mosquitoes over time (**Supplementary Figure 3**). This finding could be strengthened by including more data sets, especially on hemolymph sporozoites of control and *pycryph2* disrupted parasites.

This study revealed that PyCryPH2 localizes to the crystalloid, an organelle unique to ookinetes, and is involved in the microstructure of crystalloids. Loss of PyCryPH2 influences sporozoite maturation, leading to a deficiency in the invasion of salivary glands and the infection of mammalian livers. Our findings, taken together with previous studies, indicate that crystalloid proteins such as LAP family, DHHC10, NAD(P) transhydrogenase, and PyCryPH2 are indirectly required for sporozoite formation and/or maturation, albeit to different degrees. Elucidating the molecular mechanisms that link *pycryph2* disruption to aberrant crystalloid microstructure and subsequent impaired sporozoite maturation for invasion is a critical next step to reveal the comprehensive roles of crystalloids in malaria parasite transmission from mosquito vectors to mammals.

## Data Availability

The datasets presented in this study can be found in online repositories. The names of the repository/repositories and accession number(s) can be found below: https://www.ddbj.nig.ac.jp/, PRJDB35932.

## References

[B1] Abrahamsen M. S. Templeton T. J. Enomoto S. Abrahante J. E. Zhu G. Lancto C. A. . (2004). Complete genome sequence of the apicomplexan, *Cryptosporidium parvum*. Science 304, 441–445. doi: 10.1126/science.1094786. PMID: 15044751

[B2] Braumann F. Klug D. Kehrer J. Song G. Feng J. Springer T. A. . (2023). Conformational change of*Plasmodium *TRAP is essential for sporozoite migration and transmission. EMBO Rep. 24, e57064. doi: 10.15252/embr.202357064. PMID: 37306042 PMC10328070

[B3] Carter V. Shimizu S. Arai M. Dessens J. T. (2008). PbSR is synthesized in macrogametocytes and involved in formation of the malaria crystalloids. Mol. Microbiol. 68, 1560–1569. doi: 10.1111/j.1365-2958.2008.06254.x. PMID: 18452513 PMC2615194

[B4] Chaiyawong N. Ishizaki T. Hakimi H. Asada M. Yahata K. Kaneko O. (2022). Distinct effects on the secretion of MTRAP and AMA1 in *Plasmodium yoelii* following deletion of acylated pleckstrin homology domain-containing protein. Parasitol. Int. 86, 102479. doi: 10.1016/j.parint.2021.102479. PMID: 34628068

[B5] Claudianos C. Dessens J. T. Trueman T. H. Arai M. Mendoza J. Butcher G. A. . (2002). A malaria scavenger receptor-like protein essential for parasite development. Mol. Microbiol. 45, 1473–1484. doi: 10.1046/j.1365-2958.2002.03118.x. PMID: 12354219

[B6] Dessens J. T. Saeed S. Tremp A. Z. Carter V. (2011). Malaria crystalloids: specialized structures for parasite transmission? Trends Parasitol. 27, 106–110. doi: 10.1016/j.pt.2010.12.004. PMID: 21237711 PMC3133641

[B7] Ecker A. Bushell E. S. C. Tewari R. Sinden R. E. (2008). Reverse genetics screen identifies six proteins important for malaria development in the mosquito. Mol. Microbiol. 70, 209–220. doi: 10.1111/j.1365-2958.2008.06407.x. PMID: 18761621 PMC2658712

[B8] Ghosh A. K. Jacobs-Lorena M. (2009). *Plasmodium* sporozoite invasion of the mosquito salivary gland. Curr. Opin. Microbiol. 12, 394–400. doi: 10.1016/j.mib.2009.06.010. PMID: 19608457 PMC2759692

[B9] Ishino T. Murata E. Tokunaga N. Baba M. Tachibana M. Thongkukiatkul A. . (2019). Rhoptry neck protein 2 expressed in*Plasmodium *sporozoites plays a crucial role during invasion of mosquito salivary glands. Cell. Microbiol. 21, e12964. doi: 10.1111/cmi.12964. PMID: 30307699 PMC6587811

[B10] Ishino T. Tachibana M. Baba M. Iriko H. Tsuboi T. Torii M. (2020). Observation of morphological changes of female osmiophilic bodies prior to*Plasmodium *gametocyte egress from erythrocytes. Mol. Biochem. Parasitol. 236, 111261. doi: 10.1016/j.molbiopara.2020.111261. PMID: 31981605

[B11] Janse C. J. Ramesar J. Waters A. P. (2006). High-efficiency transfection and drug selection of genetically transformed blood stages of the rodent malaria parasite *Plasmodium berghei*. Nat. Protoc. 1, 346–356. doi: 10.1038/nprot.2006.53. PMID: 17406255

[B12] Jenwithisuk R. Kangwanrangsan N. Tachibana M. Thongkukiatkul A. Otsuki H. Sattabongkot J. . (2018). Identification of a PH domain-containing protein which is localized to crystalloid bodies of*Plasmodium *ookinetes. Malar. J. 17, 466. doi: 10.1186/s12936-018-2617-6. PMID: 30545367 PMC6291999

[B13] Kangwanrangsan N. Tachibana M. Jenwithisuk R. Tsuboi T. Riengrojpitak S. Torii M. . (2013). A member of the CPW-WPC protein family is expressed in and localized to the surface of developing ookinetes. Malar. J. 12, 129. doi: 10.1186/1475-2875-12-129. PMID: 23587146 PMC3637178

[B14] Kennedy M. Fishbaugher M. E. Vaughan A. M. Patrapuvich R. Boonhok R. Yimamnuaychok N. . (2012). A rapid and scalable density gradient purification method for*Plasmodium *sporozoites. Malar. J. 11, 421. doi: 10.1186/1475-2875-11-421. PMID: 23244590 PMC3543293

[B15] Kojin B. B. Adelman Z. N. (2019). The Sporozoite’s journey through the mosquito: A critical examination of host and parasite factors required for salivary gland invasion. Front. Ecol. Evol. 7, 284. doi: 10.3389/fevo.2019.00284

[B16] Kumar P. Tripathi A. Ranjan R. Halbert J. Gilberger T. Doerig C. . (2014). Regulation of *Plasmodium falciparum* development by calcium-dependent protein kinase 7 (PfCDPK7). J. Biol. Chem. 289, 20386–20395. doi: 10.1074/jbc.M114.561670. PMID: 24895132 PMC4106351

[B17] Lavazec C. Moreira C. K. Mair G. R. Waters A. P. Janse C. J. Templeton T. J. (2009). Analysis of mutant *Plasmodium berghei* parasites lacking expression of multiple PbCCp genes. Mol. Biochem. Parasitol. 163, 1–7. doi: 10.1016/j.molbiopara.2008.09.002. PMID: 18848846

[B18] Lemmon M. A. (2007). Pleckstrin homology (PH) domains and phosphoinositides. Biochem. Soc Symp. 74, 81–93. doi: 10.1042/BSS2007c08 PMC3777418 17233582

[B19] Lin J. W. Annoura T. Sajid M. Chevalley-Maurel S. Ramesar J. Klop O. . (2011). A novel ‘gene insertion/marker out’ (GIMO) method for transgene expression and gene complementation in rodent malaria parasites. PLOS ONE 6, e29289. doi: 10.1371/journal.pone.0029289. PMID: 22216235 PMC3246482

[B20] Lindner S. E. Swearingen K. E. Shears M. J. Walker M. P. Vrana E. N. Hart K. J. . (2019). Transcriptomics and proteomics reveal two waves of translational repression during the maturation of malaria parasite sporozoites. Nat. Commun. 10, 4964. doi: 10.1038/s41467-019-12936-6. PMID: 31673027 PMC6823429

[B21] Meis J. F. Ponnudurai T. (1987). Ultrastructural studies on the interaction of *Plasmodium falciparum* ookinetes with the midgut epithelium of *Anopheles stephensi* mosquitoes. Parasitol. Res. 73, 500–506. doi: 10.1007/BF00535323. PMID: 3321042

[B22] Nozaki M. Baba M. Tachibana M. Tokunaga N. Torii M. Ishino T. (2020). Detection of the rhoptry neck protein complex in*Plasmodium *sporozoites and its contribution to sporozoite invasion of salivary glands. mSphere 5, e00325-20. doi: 10.1128/mSphere.00325-20. PMID: 32817376 PMC7440843

[B23] Otsuki H. Yokouchi Y. Iyoku N. Tachibana M. Tsuboi T. Torii M. (2015). The rodent malaria lactate dehydrogenase assay provides a high throughput solution for *in vivo* vaccine studies. Parasitol. Int. 64, 60–63. doi: 10.1016/j.parint.2015.02.001. PMID: 25701649

[B24] Powis G. Meuillet E. J. Indarte M. Booher G. Kirkpatrick L. (2023). Pleckstrin Homology [PH] domain, structure, mechanism, and contribution to human disease. Biomed. Pharmacother. 165, 115024. doi: 10.1016/j.biopha.2023.115024. PMID: 37399719

[B26] Raine J. D. Ecker A. Mendoza J. Tewari R. Stanway R. R. Sinden R. E. (2007). Female inheritance of malarial lap genes is essential for mosquito transmission. PLOS Pathog. 3, e30. doi: 10.1371/journal.ppat.0030030. PMID: 17335349 PMC1808070

[B27] Rao P. N. Santos J. M. Pain A. Templeton T. J. Mair G. R. (2016). Translational repression of the cpw-wpc gene family in the malaria parasite Plasmodium. Parasitol. Int. 65, 463–471. doi: 10.1016/j.parint.2016.06.007. PMID: 27312996

[B28] Saeed S. Carter V. Tremp A. Z. Dessens J. T. (2010). *Plasmodium berghei* crystalloids contain multiple LCCL proteins. Mol. Biochem. Parasitol. 170, 49–53. doi: 10.1016/j.molbiopara.2009.11.008. PMID: 19932717 PMC2816727

[B29] Saeed S. Carter V. Tremp A. Z. Dessens J. T. (2013). Translational repression controls temporal expression of the *Plasmodium berghei* LCCL protein complex. Mol. Biochem. Parasitol. 189, 38–42. doi: 10.1016/j.molbiopara.2013.04.006. PMID: 23684590 PMC3694310

[B30] Saeed S. Tremp A. Z. Dessens J. T. (2015). Biogenesis of the crystalloid organelle in*Plasmodium *involves microtubule-dependent vesicle transport and assembly. Int. J. Parasitol. 45, 537–547. doi: 10.1016/j.ijpara.2015.03.002. PMID: 25900212 PMC4459735

[B31] Saeed S. Tremp A. Z. Dessens J. T. (2018). The*Plasmodium *LAP complex affects crystalloid biogenesis and oocyst cell division. Int. J. Parasitol. 48, 1073–1078. doi: 10.1016/j.ijpara.2018.09.002. PMID: 30367865 PMC6284103

[B32] Saeed S. Tremp A. Z. Sharma V. Lasonder E. Dessens J. T. (2020). NAD(P) transhydrogenase has vital non-mitochondrial functions in malaria parasite transmission. EMBO Rep. 21, e47832. doi: 10.15252/embr.201947832. PMID: 31951090 PMC7054674

[B33] Santos J. M. Duarte N. Kehrer J. Ramesar J. Avramut M. C. Koster A. J. . (2016). Maternally supplied S-acyl-transferase is required for crystalloid organelle formation and transmission of the malaria parasite. Proc. Natl. Acad. Sci. U.S.A. 113, 7183–7188. doi: 10.1073/pnas.1522381113. PMID: 27303037 PMC4932936

[B34] Schindelin J. Arganda-Carreras I. Frise E. Kaynig V. Longair M. Pietzsch T. . (2012). Fiji: an open-source platform for biological-image analysis. Nat. Methods 9, 676–682. doi: 10.1038/nmeth.2019. PMID: 22743772 PMC3855844

[B35] Sinden R. E. (2017). Developing transmission-blocking strategies for malaria control. PLOS Pathog. 13, e1006336. doi: 10.1371/journal.ppat.1006336. PMID: 28683121 PMC5500365

[B36] Suarez C. Lentini G. Ramaswamy R. Maynadier M. Aquilini E. Berry-Sterkers L. . (2019). A lipid-binding protein mediates rhoptry discharge and invasion in *Plasmodium falciparum* and *Toxoplasma gondii* parasites. Nat. Commun. 10, 4041. doi: 10.1038/s41467-019-11979-z. PMID: 31492901 PMC6731292

[B37] Sultan A. A. Thathy V. Frevert U. Robson K. J. H. Crisanti A. Nussenzweig V. . (1997). TRAP is necessary for gliding motility and infectivity of*Plasmodium *sporozoites. Cell 90, 511–522. doi: 10.1016/s0092-8674(00)80511-5. PMID: 9267031

[B38] Tachibana M. Baba M. Iriko H. Shinzawa N. Torii M. Ishino T. (2024). Identification of a novel protein localized to the crystalloid of the*Plasmodium *ookinete. Parasitol. Int. 101, 102892. doi: 10.1016/j.parint.2024.102892. PMID: 38565335

[B39] Templeton T. J. Pain A. (2016). Diversity of extracellular proteins during the transition from the ‘proto-apicomplexan’ alveolates to the apicomplexan obligate parasites. Parasitology 143, 1–17. doi: 10.1017/S0031182015001213. PMID: 26585326

[B40] Tosini F. Agnoli A. Mele R. Gomez Morales M. A. Pozio E. (2004). A new modular protein of *Cryptosporidium parvum*, with ricin B and LCCL domains, expressed in the sporozoite invasive stage. Mol. Biochem. Parasitol. 134, 137–147. doi: 10.1016/j.molbiopara.2003.11.014. PMID: 14747151

[B41] Tremp A. Z. Saeed S. Sharma V. Lasonder E. Dessens J. T. (2020). *Plasmodium berghei* LAPs form an extended protein complex that facilitates crystalloid targeting and biogenesis. J. Proteomics 227, 103925. doi: 10.1016/j.jprot.2020.103925. PMID: 32736136 PMC7487766

[B42] Tsuboi T. Cao Y. M. Hitsumoto T. Yanagi T. Kanbara H. Torii M. (1997). Two antigens on zygotes and ookinetes of *Plasmodium yoelii* and *Plasmodium berghei* that are distinct targets of transmission-blocking immunity. Infect. Immun. 65, 2260–2264. doi: 10.1128/iai.65.6.2260-2264.1997. PMID: 9169761 PMC175313

[B44] Ukegbu C. V. Gomes A. R. Giorgalli M. Campos M. Bailey A. J. Besson T. R. B. . (2023). Identification of genes required for*Plasmodium *gametocyte-to-sporozoite development in the mosquito vector. Cell Host Microbe 31, 1539–1551. doi: 10.1016/j.chom.2023.08.010. PMID: 37708854 PMC7618085

[B45] Zhang M. Kaneko I. Tsao T. Mitchell R. Nardin E. H. Iwanaga S. . (2016). A highly infectious *Plasmodium yoelii* parasite, bearing *Plasmodium falciparum* circumsporozoite protein. Malar. J. 15, 202. doi: 10.1186/s12936-016-1248-z. PMID: 27068454 PMC4828769

