# Supplementary figures and images for: A pleckstrin homology domain protein is involved in crystalloid formation in Plasmodium ookinetes and affects the maturation of infective sporozoites
Source: Front Cell Infect Microbiol. 2026 May 18;16:1777159. doi: 10.3389/fcimb.2026.1777159 (PMC13223096; doi:10.3389/fcimb.2026.1777159)

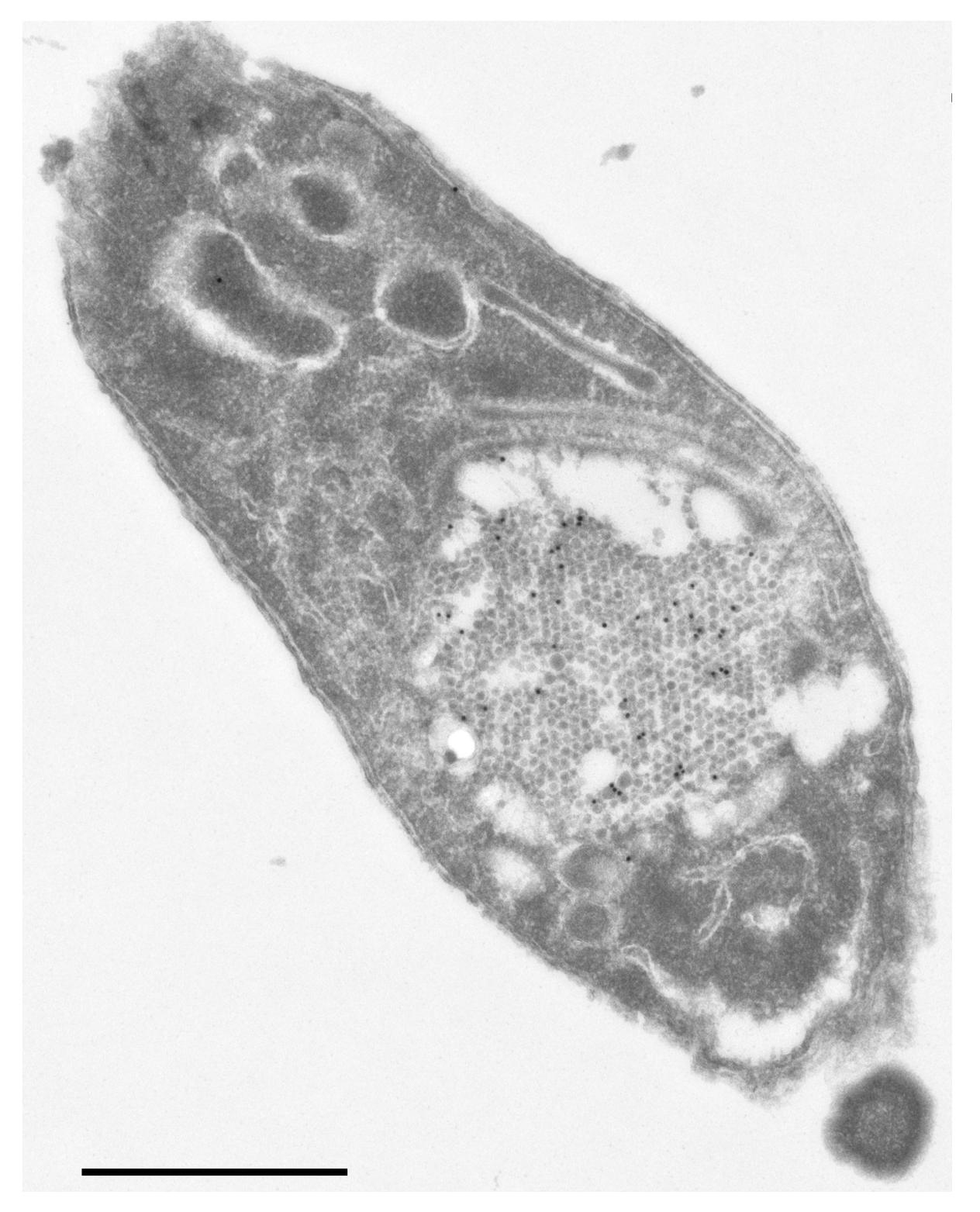

Supplement: Supplementary file 1 [file Image1.tif]

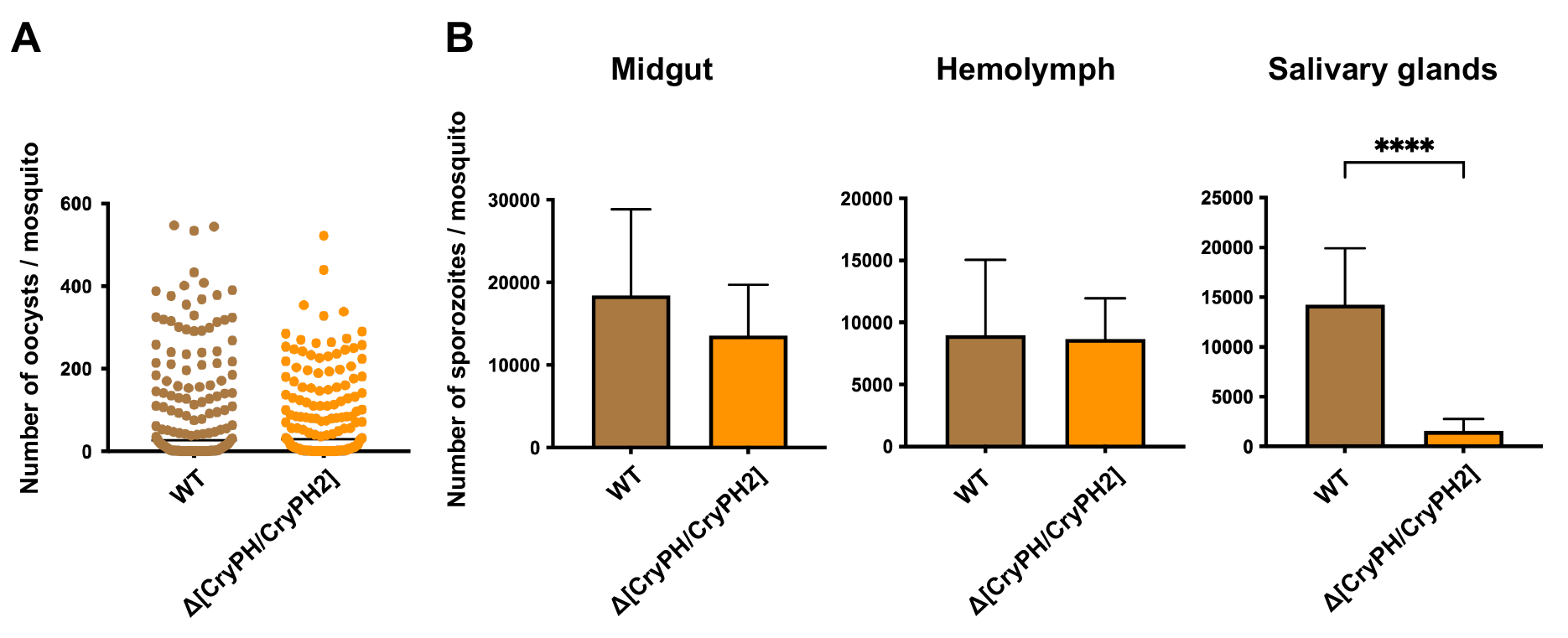

Supplement: Supplementary file 2 [file Image2.tif]

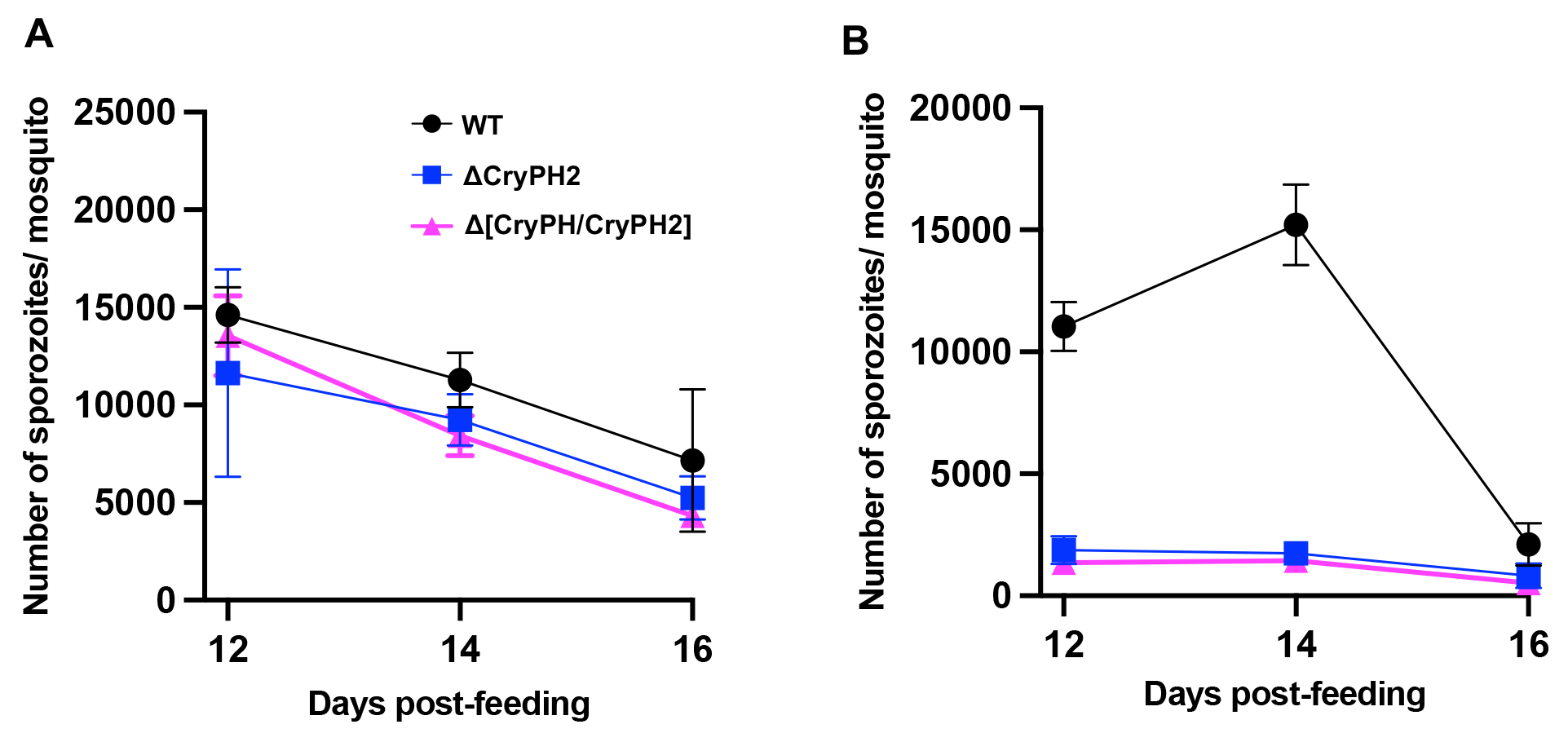

Supplement: Supplementary file 3 [file Image3.tif]

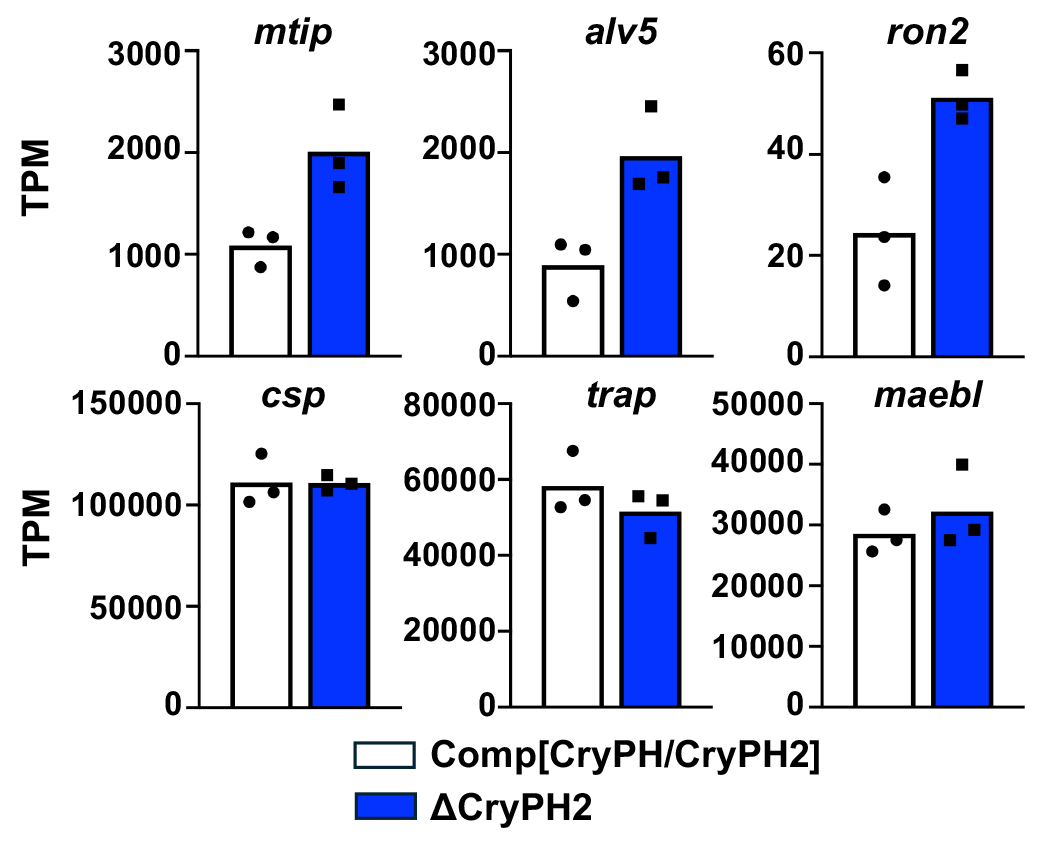

Supplement: Supplementary file 4 [file Image4.tif]
